# Antimicrobial Resistance Diversity Suggestive of Distinct *Salmonella* Typhimurium Sources or Selective Pressures in Food-Production Animals

**DOI:** 10.3389/fmicb.2019.00708

**Published:** 2019-04-12

**Authors:** Kate C. Mellor, Liljana Petrovska, Nicholas R. Thomson, Kate Harris, Stuart W. J. Reid, Alison E. Mather

**Affiliations:** ^1^Royal Veterinary College, Hatfield, United Kingdom; ^2^London School of Hygiene & Tropical Medicine, London, United Kingdom; ^3^Animal and Plant Health Agency, Weybridge, United Kingdom; ^4^Wellcome Trust Sanger Institute, Wellcome Genome Campus, Hinxton, United Kingdom; ^5^Quadram Institute Bioscience, Norwich, United Kingdom

**Keywords:** antimicrobial resistance, ecological diversity, surveillance, *Salmonella* Typhimurium, food-production animals

## Abstract

*Salmonella enterica* subsp. *enterica* serovar Typhimurium is a common cause of enterocolitis in humans globally, with multidrug resistant (MDR) strains posing an enhanced threat. *S.* Typhimurium is also a pathogen in food-production animals, and these populations can act as reservoirs of the bacterium. Therefore, surveillance and control measures within food-production animal populations are of importance both to animal and human health and have the potential to be enhanced though improved understanding of the epidemiology of *S.* Typhimurium within and between food-production animal populations. Here, data from Scotland and national surveillance England and Wales data for isolates from cattle (*n* = 1115), chickens (*n* = 248) and pigs (*n* = 2174) collected between 2003 and 2014 were analyzed. Ecological diversity analyses and rarefaction curves were used to compare the diversity of observed antimicrobial resistance (AMR) profiles between the host species, and within host species populations. Higher AMR profile diversity was observed in isolates from pigs compared to chickens across diversity measures and isolates from cattle for three of four diversity measures. Variation in AMR profile diversity between production sectors was noted, with higher AMR diversity of isolates from broiler compared to layer chickens, breeder compared to rearer and finisher pigs and beef compared to dairy cattle. Findings indicate variation in AMR profile diversity both within and between food-production animal host species. These observations suggest alternate sources of AMR bacteria and/or variation in selective evolutionary pressures within and between food-production animal host species populations.

## Introduction

Globally, drug resistant infections are projected to cause 10 million human deaths at a cost of 100 trillion USD annually by 2050 if current trends continue (O’Neill, 2014). Selection pressure caused by the widespread use of antimicrobials in human medicine, veterinary medicine and agriculture has increased the dissemination and prevalence of AMR in bacteria (Laxminarayan and Heymann, 2012). Improving our understanding of the emergence and spread of AMR bacteria within and between host species populations is essential to inform effective control policies to prevent or reduce dissemination.

In the EU, over 100,000 cases of enterocolitis, costing an estimated €3 billion annually, are attributed to non-typhoidal *Salmonella* infections, of which *Salmonella enterica* subsp. *enterica* serovar Typhimurium is the second most common serovar (European Food Safety Authority and European Centre for Disease Prevention and Control, 2017). *Salmonella* Typhimurium is a zoonotic pathogen and the primary reservoir is thought to be food-production animals, with the majority of human cases deriving through the food chain (Majowicz et al., 2010), although more recent studies have suggested a more nuanced situation (Mather et al., 2013). Compared to antimicrobial sensitive strains, those resistant to therapeutically relevant antimicrobials pose a greater threat to public health because they are associated with higher morbidity and mortality rates (Helms et al., 2002; Cosgrove, 2006; Depuydt et al., 2008). Multi-drug resistant (MDR) strains, most notably definitive type (DT)104, have been documented to disseminate globally, causing infections in multiple host species including humans and food-production animals (Leekitcharoenphon et al., 2016).

National surveillance of *S.* Typhimurium in United Kingdom animal populations is conducted primarily for outbreak identification control purposes to limit the public health risk. Mandatory active surveillance is conducted for poultry, whilst passive surveillance is conducted for other food producing animals (DEFRA, 2007, 2008, 2009). Passive surveillance relies upon submission of samples by veterinarians or farmers for clinical diagnostic purposes. Identification of *Salmonella* from an animal source is reportable to the APHA where confirmation and phenotypic antimicrobial susceptibility testing and phage typing are conducted (Zoonoses Order, 1989; Animal and Plant Health Agency, 2017). Phenotypic AMR profile may not consistently correlate with bacterial genetic lineage, due to the importance of horizontal gene transfer for the transmission of AMR, and phage types can be polyphyletic (Chen et al., 2005; Petrovska et al., 2016). The resolution afforded through analysis of phage type and AMR phenotypic profiles is therefore restricted, but the combination of phage type and phenotypic AMR profile have proved useful for distinguishing between isolates for outbreak detection purposes.

Variation in the prevalence of resistance of bacterial pathogens to individual antimicrobials between food-production animal groups has been attributed to differences in selective pressures between production systems and dissemination of AMR clones, e.g., DT104. However, our understanding of the evolution and dissemination of resistant bacteria within and between food-production animal populations is limited (Young et al., 2009). AMR profiles unique to individual host species would not be expected if there was transfer of strains between host species populations. The rate of transfer of strains between host species could be influenced by ecological or pathogen related barriers which could result in differences in the observed AMR profiles between host species (Rabsch et al., 2002). The presence of shared AMR profiles between multiple host species can arise through a common source of infection, transmission of bacteria or mobile genetic elements (MGEs) carrying AMR genes between the host species populations or through independent emergence or acquisition of resistance determinants.

Opportunities for transfer of non-host-restricted strains between host species are numerous and include mixed-species farms, markets, common grazing, contaminated feed products and movements of fomites, personnel or wildlife (Davies and Wray, 1997; Robinson and Christley, 2007; Skov et al., 2008). The risk of transfer between host species is likely to vary between food-production animal species due to the differences in industry structure and standard biosecurity protocols (Lindström et al., 2012). Enhanced surveillance and control measures for United Kingdom poultry through the National Control Program (NCP) were introduced to reduce the prevalence of *Salmonella* Typhimurium and *Salmonella* Enteritidis on farms (DEFRA, 2007, 2008, 2009). Combined with biosecurity practices and a pyramidal industry structure, the United Kingdom *S.* Typhimurium population in chickens could be predicted to be relatively isolated from *S.* Typhimurium circulating in other host species. However, free-range poultry could be exposed to *Salmonella* transferred by wild birds and flies (Wales et al., 2010; Andrés et al., 2013).

Variation in AMR profile diversity within or between host species could indicate a greater diversity of AMR bacteria sources entering a host species population or indicate continuous evolution in the host population in response to greater selective pressures. Additionally, distinct differences in AMR profiles between host species could indicate epidemiological barriers to transmission. Here, the available national surveillance data for *S.* Typhimurium isolates, collected over an 11-year period, have been used to determine whether or not there are detectable differences in circulating AMR profiles within and between *S.* Typhimurium isolates from cattle, chicken and pig populations.

## Materials and Methods

### Data

Routine surveillance samples from cattle, chicken and pigs submitted to the APHA between 2003 and 2014, which had a recorded AMR profile, were eligible for inclusion in the study. The majority of samples were from England, with the remaining samples from Wales (*n* = 168, 4.8%) and Scotland (*n* = 40, 1.1%). United Kingdom region information was unavailable for 230 isolates (6.5%). *Salmonella* is a reportable pathogen in both animals and humans in the United Kingdom; samples are typically submitted for clinical diagnostic purposes to regional laboratories. Samples from chickens are also collected through the National Control Program which requires sampling of commercial flocks at predetermined points in the production cycle (DEFRA, 2007, 2008, 2009). Due to the nature of the sample collection, NCP isolates are likely to be more representative of the microbial population in healthy birds compared to passive surveillance isolates obtained from symptomatic birds. Samples from food-producing animals which test positive for *Salmonella* are submitted to the APHA for confirmation and antimicrobial susceptibility testing in accordance with the Zoonoses Order (1989).

At the APHA, phage-typing was conducted (Anderson et al., 1977) and antimicrobial susceptibility testing performed using a disk diffusion technique (Animal and Plant Health Agency, 2017). Antimicrobials tested and breakpoints for classification as sensitive or resistant are detailed in Table 1. Both breakpoints and antimicrobial disk concentrations changed during the period 2003 to 2014 for multiple antimicrobials. Up to 2007 a 13 mm breakpoint was used, with classification as resistant if growth inhibition zone ≤ 13 mm. British Society for Antimicrobial Chemotherapy (BSAC) recommended breakpoints were adopted for amikacin, cefotaxime, ceftazidime and ciprofloxacin from 2007 onward, and subsequently for gentamicin, sulphamethoxazole/trimethoprim, amoxicillin/clavulanic acid and chloramphenicol (British Society for Antimicrobial Chemotherapy, 2013). The historical veterinary breakpoints were used for the remaining antimicrobials, for which no BSAC breakpoints are available, throughout the period of study (Veterinary Medicines Directorate, 2013). Additionally, some changes to antimicrobials tested were made, these are detailed in Table 1.

**Table 1:** Antimicrobials used for resistance phenotyping of *Salmonella* Typhimurium isolates at the Animal and Plant Health Agency between 2003 and 2014.

				
Antimicrobial class	Antimicrobial	Concentration (μg per ml)	Zone size (resistant if <x mm)	BSAC cut-off introduction
Aminoglycoside	Neomycin	10	13	–
	Amikacin	30	18	2007
	Gentamicin	10	19	2008
	Streptomycin°	10	13	–
	Apramycin	15	13	–
Cephalosporin (3rd generation)	Cefoperazone^∗^	30	13	–
	Cefotaxime^∗^	30	29	2007
	Ceftazidime	30	26	2007
Quinolone	Nalidixic acid	30	13	
	Ciprofloxacin^♢^	1	19	2007
Folate pathway inhibitor	Sulphamethoxazole/trimethoprim	25	15	2008
	Sulphonamide compounds	300	13	–
Nitrofuran	Furazolidone	15	13	–
Penicillin	Ampicillin	10	13	–
	Amoxicillin/clavulanic acid	30	14	2008
Phenicol	Chloramphenicol^+^	30	20	2008
Tetracycline	Tetracycline	10	13	–
Polymyxin	Colistin^•^	25	13	–


*Antimicrobial susceptibility testing was performed using the British Society for Antimicrobial Chemotherapy disk diffusion technique, with isolates classified as resistant or sensitive to the antimicrobial according to the zone size cut-off values. ^∗^Testing for sensitivity to cefoperazone was replaced with cefotaxime in 2004. ^+^10 μg until 2008. ^♢^from 2004 onwards. ^•^2003 only. °25 μg from 2003 to 2008. Until 2007 a 13 mm breakpoint was used, with classification if growth inhibition zone ≤ 13 mm, BSAC recommended breakpoints were introduced for amikacin, cefotaxime, ceftazidime and ciprofloxacin from 2007 onward, and for gentamicin, sulphamethoxazole/trimethoprim, amoxicillin/clavulanic acid and chloramphenicol from 2008 onward. The historical veterinary breakpoints were used for the remaining antimicrobials throughout the period of study*.

### Diversity Analysis

Ecological measures of AMR phenotype diversity were calculated in R (R Core Team, 2016) using the “Vegan” package (Oksanen et al., 2011). Similar to the analysis described by Mather et al. (2012), four diversity measures were calculated: SR, SE, SD and reciprocal BP with 95% confidence intervals generated through resampling. Each measure differentially weights the importance of SR and species abundance. In this study, a “species” is defined as a unique AMR profile, including the profile corresponding to susceptibility to all tested antimicrobials. The AMR profile (or antibiogram) of an isolate is the combination of AMR phenotype (susceptible or non-susceptible) to each drug tested. SR reflects the richness of AMR profiles without weighting of abundance. SE and SD are measures in which both SR and relative abundance are taken into account. The BP diversity index is related to the proportion of isolates with the most common AMR profile.

Changes in antimicrobial testing protocol could impact upon results, particularly where the proportion of isolates from each host vary between time periods with different testing protocols. Four different ‘testing periods’ were identified within which antimicrobial sensitivity testing protocol was consistent (2003; 2004–2006; 2007; 2008–2014). As calculated diversity indices are dependent on sample size, for each ‘testing period’ the host species groups with larger sample sizes were randomly subsampled to the size of the smaller host species group. For each host species the combined data for the four testing periods was used as the input for the diversity analyses. Following 10,000 iterations the mean of iterations and confidence intervals of diversity indices were then calculated. The exponent of SE values and the reciprocal of SD and BP values were calculated to convert diversity indices into the effective number of profiles prior to plotting of results. The diversity measures were deemed to differ if the 95% confidence intervals of the values of two host species did not overlap.

Diversity analyses were conducted to compare between host species, and within host species based upon production type metadata.

### Network Analysis

The network of connectivity of AMR profiles in the host species was visualized using the “igraph” package (Csárdi and Nepusz, 2006) in R. The edges represent where a single change in the AMR profile occurred between two AMR profiles (nodes). Nodes are colored according to the host species, or host species combination.

### Rarefaction Curves

Rarefaction analyses were performed using the “iNEXT” package in R (Hsieh et al., 2018), which examines the SR of phenotypic profiles at each sample size. The rarefaction curves enable evaluation as to whether the total AMR profile diversity was captured by the sampling, and comparison of AMR profile diversity between host species. To account for changes in antimicrobial sensitivity testing procedure data is presented for the four time periods for which testing procedures were identical across the host species.

### Common AMR Profiles and Associated Phage Types

For each of the individual host species, the top five most frequent AMR profiles, and association with phage types (most common and total number) were determined and compared between host species.

### Compare AMR Profiles Between Host Species

All analyses were processed in R. To visualize AMR profiles common to multiple hosts and unique to individual hosts, the “eulerr” package was used to create a proportional Venn diagram of ellipses (Larsson, 2018).

### Comparison of Observed and Expected Numbers of AMR Profiles Shared by All Host Species

The expected number of AMR profiles shared by all host species was compared to the observed number. A subsample to the size of the smallest host species group for each ‘testing period’ without replacement was generated for each host species and data joined prior to source randomization for each isolate without replacement and the number of AMR profiles common to all host species recorded of the 10,000 iterations. The observed distribution was generated without source randomization. The observed number of phenotypic AMR profiles common to all host species groups (mean of 10,000 iterations with subsampling) was considered to be significantly different to the expected number if falling in the last or first 2.5th percentile of the distribution of expected number, equivalent to a two tailed at *p* < 0.05.

### Comparison of Observed and Expected Numbers of AMR Profiles Unique to Individual Host Species

The distributions of observed numbers of AMR profiles unique to each host were generated using subsampling to the smallest host species group for each time period without replacement. The number of AMR profiles unique to individual hosts were recorded for each of the 10,000 iterations.

## Results

Between 2003 and 2014 a total of 3537 isolates (1115 from cattle, 248 from chickens and 2174 from pigs) were submitted to the APHA. The majority (95.8%) of submissions were obtained though passive surveillance with the remainder obtained through the NCP active surveillance system for poultry. Phenotypic antimicrobial test data were available for all except one isolate which was excluded from analysis. The data for these 3537 samples were used for the general analysis. A summary table of the antimicrobial resistance profiles observed in each host species is available in Supplementary Table 1. Production level data were available for 920 (82%) of isolates from cattle, 233 (94%) of isolates from chickens and 1180 (54.3%) of isolates from pigs.

Some isolates were collected on the same day from the same farm, as indicated by a shared submission reference. The proportions of isolates with shared submission reference varied between the host species 24.6% (274/1115) for cattle, 26.6% (66/248) for chickens and 48.9% (1062/2174) for pigs. The submission references indicate whether multiple isolates have been collected from the same farm on the same day, however, whether the isolates are from the same animal group or different animal groups on the farm is unknown. Multiple AMR profiles of isolates with a shared submission reference have been observed and these samples may have been taken from different animal groups (Table 2), however, this metadata was not available for analysis in this study. Isolates with the same submission reference which share an AMR profile could be the same strain, however, this cannot be determined given the resolution of the data.

**Table 2:** Isolates with shared submission references indicating collection at the same farm location on the same day.

Host species	Number (percentage) of isolates with shared submission reference	Number of AMR profiles associated with a single submission reference			
		
		1	2	3	4
Cattle	274 (24.6)	88	13	1	0
Chicken	66 (26.6)	25	2	0	0
Pig	1062 (48.9)	198	75	18	3


### Comparison of AMR Profiles Between Host Species

**FIGURE 1:**
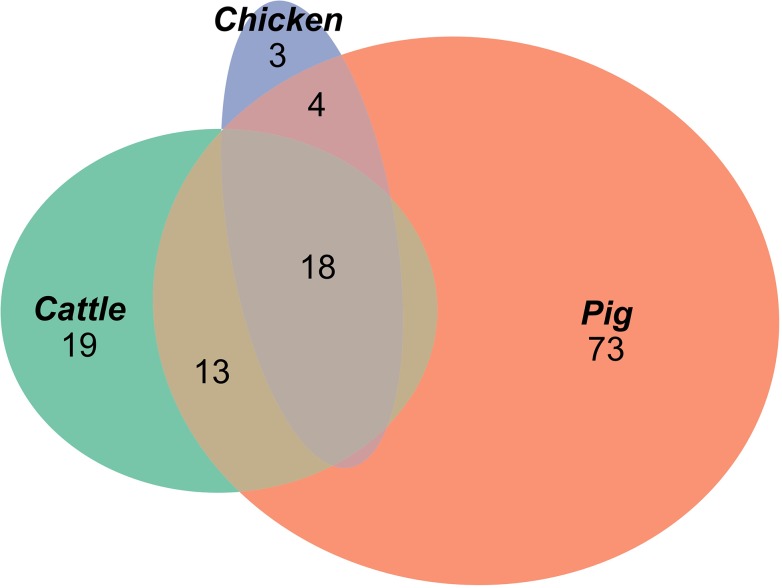
Proportional elliptical Venn diagram of AMR profiles of isolates from pig, cattle and chicken sources.

**FIGURE 2:**
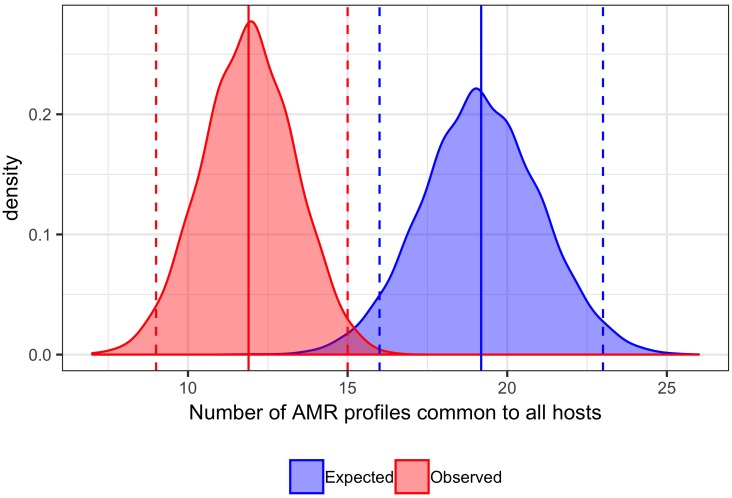
Comparison of expected and observed number of unique AMR profiles common to cattle, pig and chicken host species. Distribution of 10,000 permutations of number of AMR profiles shared by all host species. Blue dashed vertical lines: first and last 2.5th percentiles of expected values (16,23). Blue solid vertical line: mean expected number of AMR profiles common to all hosts (19.19). Red dashed vertical lines: first and last 2.5th percentiles of observed values (9,15). Red solid vertical line: mean expected number of AMR profiles common to all hosts (11.87). Bandwidth 0.5.

A total of 129 AMR profiles, including full susceptibility, were observed amongst isolates from cattle, pigs and chickens, 18 (14%) of which were observed in all three host species groups; these 18 AMR profiles represented 89.9% of isolates from cattle, 96.8% of isolates from chickens and 76.6% of isolates from pigs (Figure 1). Separation between observed and expected distributions of AMR profiles common to all host species was observed (Figure 2).

**FIGURE 3:**
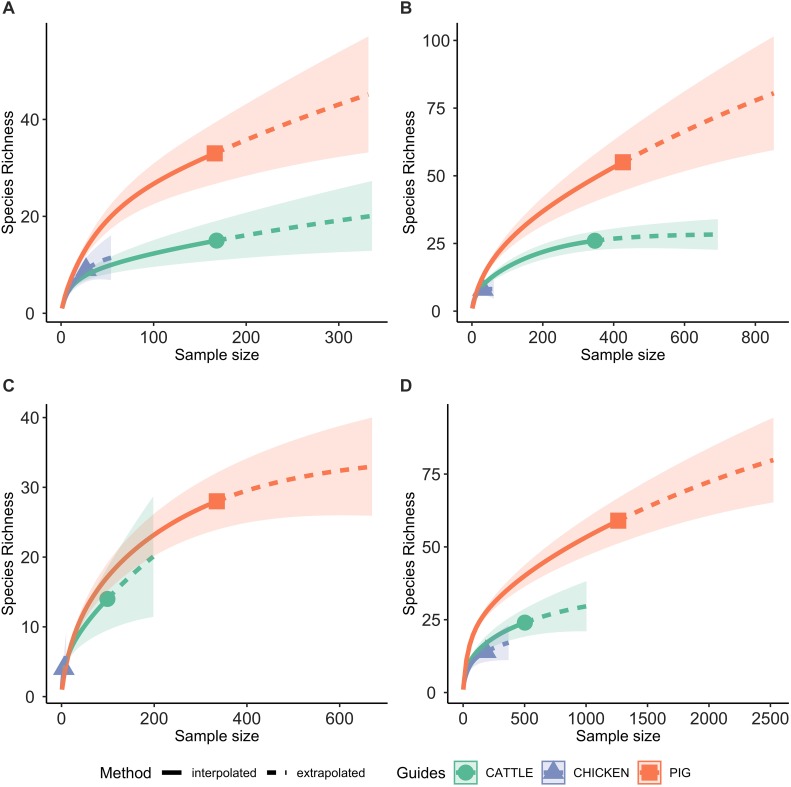
Rarefaction curves, with 95% confidence intervals, of the number of phenotypic AMR profiles for each host species (species richness). Extrapolation to two times the sample size for each host species. **(A)** 2003 only; **(B)** 2004–2006 only; **(C)** 2007 only; **(D)** 2008–2014 only.

**FIGURE 4:**
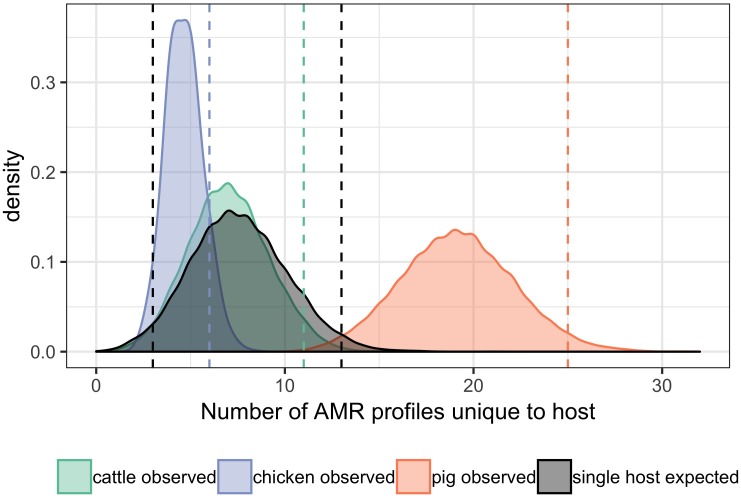
Differences in the observed number of AMR profiles unique to individual hosts compared with number expected for a single host. Bandwidth = 0.5. Vertical dashed orange lines: first and last 2.5th percentiles of values of pig observed data. Vertical dashed green line: last 2.5th percentile of values of cattle observed data. Vertical dashed blue line: last 2.5th percentile of values of chicken observed data. Vertical dashed black lines: first and last 2.5th percentiles of expected values for a single host. The lower 2.5th percentile vertical line of observed values from cattle and chickens overlap with lower 2.5th percentile vertical line for expected values for a single host.

A total of 95 profiles were observed to be unique to a single host species. Comparison of the absolute number of observed profiles between host species groups does not account for differences in sample size for each host species group. Rarefaction curves were therefore used to compare SR of phenotypic AMR profiles between host species (Figure 3), showing a higher diversity of profiles observed from pigs compared to chickens despite the low sample number from chickens. The rarefaction curves also indicate that the full AMR profile diversity of isolates from cattle are closer to being captured than for isolates from pigs. The percentage of isolates with an AMR profile unique to the host species was almost 10-fold higher in isolates from pigs compared to chickens (Table 3). After controlling for sample size, distinct separation of the observed distribution of AMR profiles unique to individual hosts can be seen between isolates from chickens and pigs but overlap with distributions of observed number of AMR profiles of isolates from cattle and expected number of AMR profiles for a single host species (Figure 4).

**Table 3:** Summary of prevalence of resistance to one or more antimicrobial, multi-drug resistant (MDR), and comparison of AMR profiles observed for each host species.

Host species	Number (%) of isolates		Observed number of AMR profiles		Number (%) of isolates with AMR profile	
			
	Resistant to one or more antimicrobial	MDR	Total	Unique to host species	Unique to host species	Common to all host species
Cattle	952 (85.4)	833 (74.7)	50	19	51 (4.6)	1003 (89.9)
Chicken	130 (52.4)	110 (44.4)	25	3	4 (1.6)	240 (96.8)
Pig	2132 (98.1)	1976 (90.9)	108	73	346 (15.9)	1665 (76.6)


*MDR is defined as phenotypic resistance to three or more classes of antimicrobials*.

Three out of the five most common AMR profiles are shared by all host species groups (Table 4). Apart from sensitive isolates, all top five most common AMR profiles include tetracycline resistance. The remainder of the profiles are resistance to ampicillin, streptomycin, sulphonamides and tetracycline ± resistance to chloramphenicol and/or trimethoprim-sulphonamides.

**Table 4:** Top five most frequent AMR profiles for each host species.

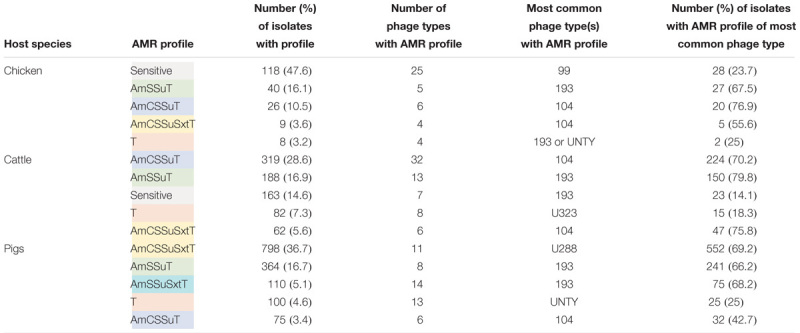

*Am, ampicillin; C, chloramphenicol; S, streptomycin; Su, sulphonamides; Sxt, trimethoprim-sulphonamides; T, tetracycline. Sensitive, sensitive to all tested antimicrobials. AMR profiles are colored*.

A greater proportion of isolates (42.7–79.8%) with a common MDR AMR profile, defined as resistance to three or more antimicrobial classes, are associated with a single phage type compared to isolates resistant to tetracycline only or sensitive to all tested antimicrobials where ≤ 25% of isolates are associated with a single phage type (Table 4). The AMR profile and most commonly associated phage type combination was consistent across host species for some AMR profiles (AmCSSuT and AmSSuT), but not others (AmCSSuSxtT and T). The most common phage type associated with the AmCSSuT profile was DT104 across all host species groups, although the percentage of isolates classified as DT104 varied from 42.7% of isolates from pigs to 76.9% of isolates from chickens. Phage type DT193 was associated with 2/5 of the most frequent AMR profiles for each of the food-production animal species groups, however, the associated AMR profiles varied between food-production animal species.

### Network Analysis

**FIGURE 5:**
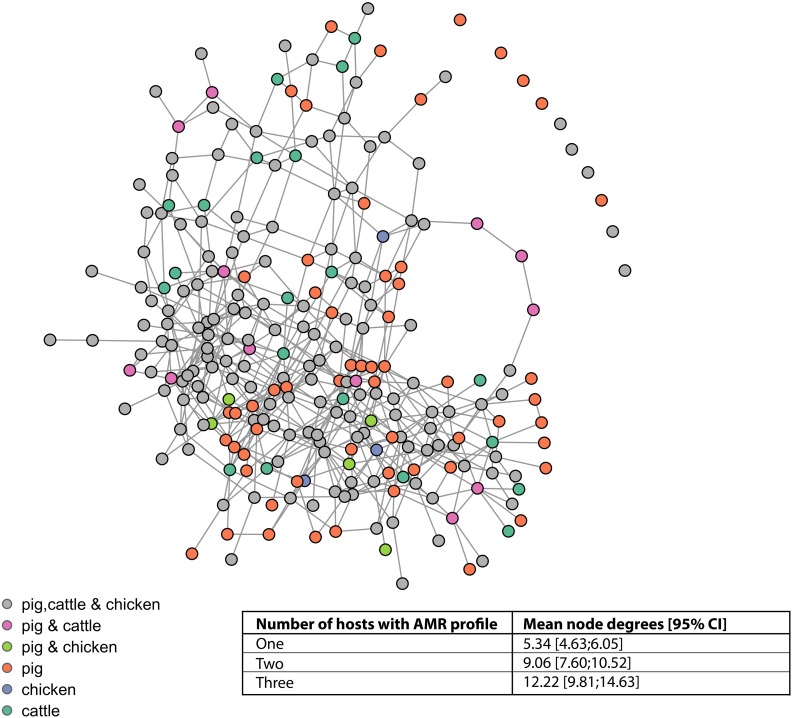
Network connectivity diagram of phenotypic antimicrobial resistance profiles. Nodes represent phenotypic antimicrobial resistance profiles. Edges represent loss or acquisition of resistance to a single antimicrobial. Inset table: mean node degrees and betweenness scores for AMR profiles observed in one, two, or three host species.

The vast majority of AMR profiles are connected in a single complex (87.6%, 113/129), a further seven isolates form a small complex, two isolates are connected to one another, and seven (of which six are from pigs) isolates are not connected to any other isolate (Figure 5). The mean node degrees were significantly higher for AMR profiles observed in multiple host species, compared to AMR profiles observed in a single host species (Inset table, Figure 5).

### Ecological Diversity Analysis

#### Diversity of AMR Profiles of Isolates From Cattle, Chicken and Pig Host Species

**FIGURE 6:**
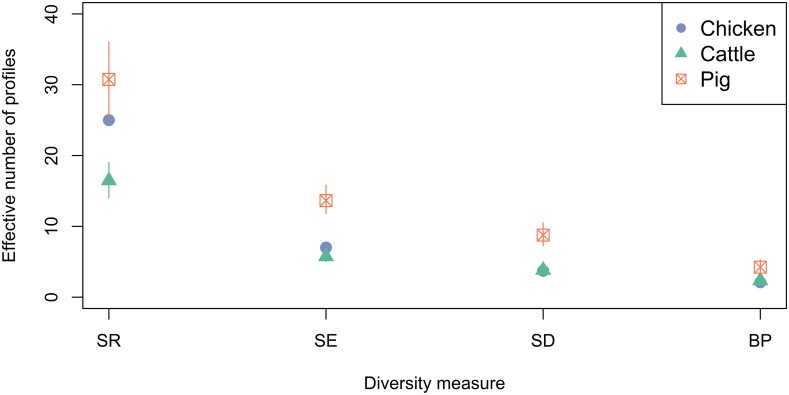
Observed ecological diversities of phenotypic antimicrobial resistance profiles of *S.* Typhimurium isolates taken from cattle, chickens and pigs between 2003 and 2014. 95% confidence intervals for passive surveillance group indices are indicated by vertical lines.

Ecological diversity calculations were performed to compare the diversity of phenotypic AMR profiles of isolates from cattle, pig and chickens (Figure 6). Across the diversity measures (SR, SE, SD, and BP) isolates from pigs had higher AMR profile diversity than those from cattle or chickens. The SR of AMR profiles of chickens isolates from was greater than isolates from cattle.

#### Diversity of AMR Profiles of Isolates From Broiler and Layer Chickens

**FIGURE 7:**
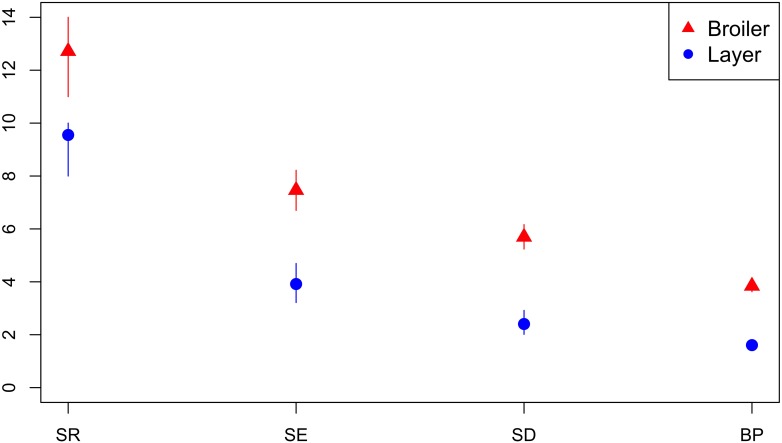
Observed ecological diversities of phenotypic antimicrobial resistance profiles of *S.* Typhimurium isolates from broiler and layer chickens.

A total of 41 isolates from breeders, 68 isolates from broilers, 112 isolates from layers and 27 isolates from an unspecified production type were submitted. The diversity of AMR profiles of isolates from broilers and layers were compared. Across all diversity measures, the effective number of AMR profiles of isolates from broilers were higher than for isolates from layer chickens (Figure 7). Six AMR profiles were common to isolates from both broilers and layers, eight AMR profiles from broilers only and three AMR profiles identified in layers only. The percentage of isolates sensitive to all 16 tested antimicrobials was almost three-fold higher (66.1%) for isolates from layers compared to isolates from broilers (23.5%).

#### Diversity of AMR Profiles of Isolates From Breeding, Rearing and Finishing Pigs

**FIGURE 8:**
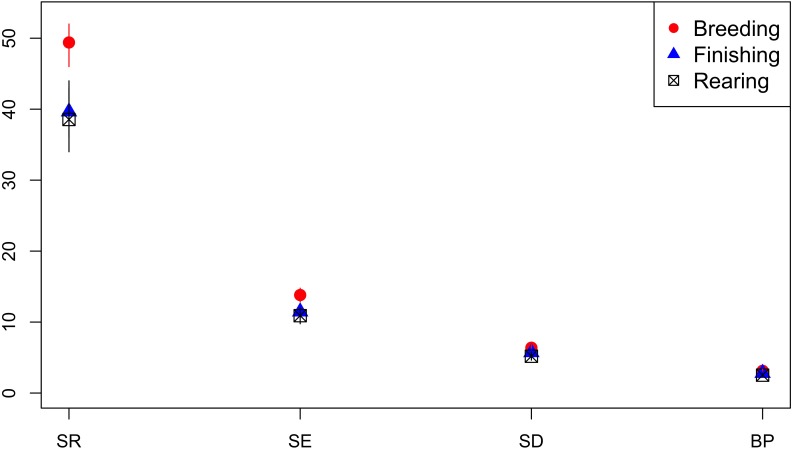
Observed ecological diversities of phenotypic antimicrobial resistance profiles of *S.* Typhimurium isolates from breeder, finisher, and rearer pigs.

Isolates from breeding pigs had a higher diversity of AMR profiles compared to isolates from rearing pigs across all diversity measures, and compared to isolates from finishing pigs for SR and Shannon Entropy diversity measures (Figure 8). Analysis was based on 548 isolates from breeders, 595 from finishers, and 737 from rearing pigs.

#### Diversity of AMR Profiles of Isolates From Dairy and Beef Cattle

**FIGURE 9:**
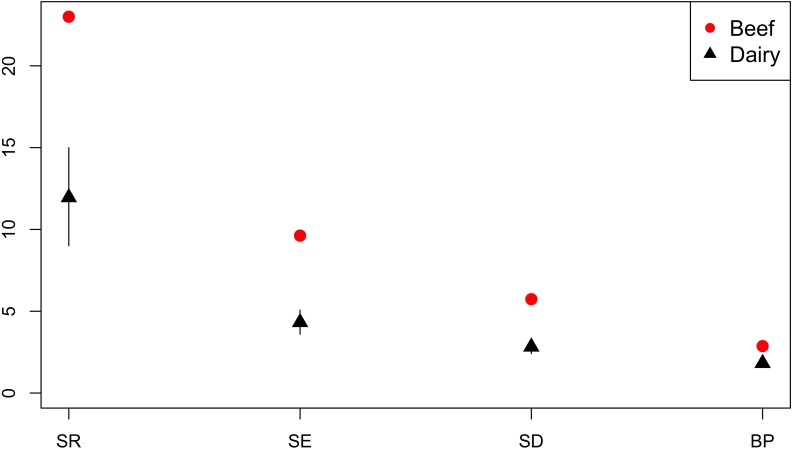
Observed ecological diversities of phenotypic antimicrobial resistance profiles of *S.* Typhimurium isolates from beef and dairy cattle.

Production sector data was available for the majority of isolates from cattle; *n* = 259 isolates were from beef cattle, and *n* = 808 isolates were from dairy cattle. Across the diversity measures (SR, SE, SD, and BP) AMR profiles of isolates from beef cattle were more diverse than isolates from dairy cattle (Figure 9).

## Discussion

*Salmonella* Typhimurium is a common cause of foodborne illness globally, with MDR strains of particular public health concern due to association with poorer patient outcomes. Reduction of prevalence in the food-production animal reservoir is important for both animal and human health and improving understanding of *S.* Typhimurium epidemiology including AMR dynamics in the food-production animal populations and has the potential to inform interventions. Using national surveillance data collected over an 11-year time period, AMR profiles of *S.* Typhimurium were compared within and between food-production animal host species populations, as variation in observed AMR profiles and AMR profile diversity could indicate variation in AMR dynamics between the host species.

Active surveillance samples from chickens from 2007 onward were included, however, the majority of samples were obtained through passive surveillance and the data are therefore biased toward strains causing clinical disease in food-production animals or identified through diagnostic testing for other diseases. Asymptomatic/subclinical infection is common in some animal species and the full diversity of circulating *S.* Typhimurium at the population level is therefore unlikely to be captured (Rostagno et al., 2012; Troxell et al., 2015). Submissions are also influenced by variation in farmer and veterinarian behavior (Schwaber et al., 2004; Laupland et al., 2007; Rempel and Laupland, 2009). Passive surveillance systems have been found to capture greater diversity of rare AMR profiles of *Salmonella* in food-production animals in Canada (Mather et al., 2016), and clinical isolates from food-production animals have been observed to have higher diversity of AMR phenotypes compared to asymptomatic food-production animals (Perron et al., 2007; Afema et al., 2015). Utilizing the national surveillance data should enable meaningful comparison of AMR profile diversity between and within food-production animal populations, although variations in surveillance between host populations could also contribute to any observed differences.

Ecological diversity analyses revealed variation in observed AMR profile diversity both between host species and between production types for chickens and pigs. The AMR profile diversity of isolates from pigs was greater than for isolates from chickens and cattle across SE, SD and BP diversity measures. The percentage of isolates resistant to one or more antimicrobials is almost two-fold higher for pigs (98.1%) compared to chickens (52.4%). This may in part account for the higher ecological diversity measures of isolates from pigs compared to chickens. The observation of 15.9% of isolates from pigs having an AMR profile unique to pigs suggests that some strains causing clinical disease in pigs are either not present, do not persist or are not causing clinical disease in cattle or chicken populations. The distinct separation of the distribution of the observed number of AMR profiles unique to pigs from that of chickens, controlling for sample size, indicates this finding is unlikely due to differential sampling intensity. Inference is limited due to the nature of passive surveillance; however, these observations suggest alternate sources of AMR bacteria and/or variation in selective evolutionary pressures between host species populations.

The separation of expected and observed distributions of AMR profiles shared by all food-production animal populations indicates that interchange of *S.* Typhimurium strains between host species is not complete, with differences in the circulating *S.* Typhimurium population between host species groups. Variation in AMR profile diversity between host species could be attributable to differences in observed lineages between host species. Bacterial typing information was limited to phage type, which affords limited resolution and does not necessarily correlate with lineage; multiple lineages can have the same phage type and AMR profile combination, and phage switching can occur (Baggesen et al., 2010; Pang et al., 2012). The consistency in AMR profile AmCSSuT and phage type DT104 combination could indicate the presence of a common single lineage between multiple host species; this particular AMR profile is also known to be chromosomally encoded in DT104 (Boyd et al., 2001). However, the most common phage type was inconsistent across host species for other AMR profiles including T and AmCSSuSxtT, suggesting different genetic lineages with the same phenotypic AMR profile may be circulating in different host species.

The selective pressures driving the maintenance of high proportions of MDR *S.* Typhimurium are not well understood and may not be due to ongoing selective pressure of antimicrobial use in food-production animal populations alone (Davis et al., 2002). Several factors, in addition to antimicrobial use, may influence variation in *S.* Typhimurium between host species including host immunity, vaccination status, biosecurity and industry structure. Vaccination can result in reduction in *S.* Typhimurium prevalence, reducing the risk of selection of AMR strains (Dagan and Klugman, 2008). Lower AMR profile diversity of isolates from chickens compared to from cattle and pigs could be expected due to widespread vaccination of laying poultry (>85%) and broiler-breeder flocks against *S.* Typhimurium, maintenance of *Salmonella* free grandparent and parent flocks and a stringent active NCP with surveillance and control measures to contain outbreaks (DEFRA, 2007, 2008, 2009; British Egg Industry Council, 2013).

Investigation of association between AMR profile diversity and risk factors within host species populations were restricted by the metadata available for retrospective analysis and therefore limited to high level observations. Higher AMR diversity was observed in isolates from breeder compared to rearer and finisher pigs. No isolates from piglets or at farrowing stage were available. This is consistent with previous findings of acquisition of AMR with successive production stages and higher pulse-field gel electrophoresis cluster diversity of monophasic *S.* Typhimurium in sows compared to piglets (Fernandes et al., 2016). Compared to layer chicken flocks, broilers were observed to have higher AMR diversity. The relatively high percentage of isolates from layers sensitive to all tested antimicrobials compared to isolates from broilers contributes to the lower diversity of AMR profiles in isolates from layers. Stringent restrictions on antimicrobial use in layer poultry (NOAH, 2016), may place greater emphasis on the need to prevent incursion of infection and reduce selective pressure for AMR to a wider range of antimicrobials. In addition, the high vaccination rates of laying poultry (British Egg Industry Council, 2013) and host genetic differences (Lumpkins et al., 2010) may contribute to variation in AMR profile diversity between the poultry sectors.

Animals are a potential reservoir for AMR bacteria and AMR genes of public health concern, therefore it is important to understand the trends of AMR in animal populations. Despite the limited discriminatory ability of phenotypic AMR profiles and phage type, variation in AMR dynamics of *S.* Typhimurium were observed within and between host species populations. The transition to whole genome sequencing technology by the APHA provides an enhanced utility for surveillance over time and space, and importantly an opportunity for improved understanding of AMR dynamics between food-production animal populations and humans at the national level. If combined with enhanced metadata, this could ultimately result in higher food security by identifying where intervention strategies may be most effectively applied and provide the opportunity for delivery of an enhanced One Health strategy.

## Ethics Statement

Data was collected through routine national surveillance and therefore did not require study permissions.

## Author Contributions

KM and AM contributed to the design of the study. KM performed the analyses. KH and LP were responsible for provision of data. KM wrote the first draft of the manuscript. All authors contributed to manuscript revision, read and approved the submitted version.

## Conflict of Interest Statement

The authors declare that the research was conducted in the absence of any commercial or financial relationships that could be construed as a potential conflict of interest.

## Abbreviations

AMR: antimicrobial resistance
APHA: Animal and Plant Health Agency
BP: Berger Parker
MDR: multi-drug resistance
NCP: National Control Program
SD: Simpsons diversity
SE: Shannon entropy
SR: species richness.
