# Supplementary material for: Antimicrobial Resistance Diversity Suggestive of Distinct Salmonella Typhimurium Sources or Selective Pressures in Food-Production Animals
Source: Front Microbiol. 2019 Apr 12;10:708. doi: 10.3389/fmicb.2019.00708 (PMC6473194; doi:10.3389/fmicb.2019.00708)
Supplement: Supplementary file 1 [file Table_1.DOCX]

**Supplementary Table 1. Antimicrobial resistance profiles observed in each host species.** Am = ampicillin; Amc= amoxicillin/clavulanic acid; Apr= apramycin; C = chloramphenicol; Caz= ceftazidime; Cf= cefoperazone; Cip= ciprofloxacin; Cn= gentamicin; Cx=cefotaxime; Fr= furazolidone; N= neomycin; Nal= nalidixic acid; S = streptomycin; Su = sulphonamides; Sxt = trimethoprim-sulphonamides; T = tetracycline.

|  | CATTLE | CHICKEN | PIG |
| --- | --- | --- | --- |
| Am | 1 | 0 | 2 |
| AmAmcCSSuSxtT | 0 | 0 | 1 |
| AmAmcCSSuT | 0 | 2 | 0 |
| AmAmcSSuT | 0 | 0 | 10 |
| AmAprCCazCnSSuSxtT | 0 | 0 | 4 |
| AmAprCCfCnNalSuSxtT | 0 | 0 | 1 |
| AmAprCCfCnSSuSxtT | 0 | 0 | 1 |
| AmAprCCipCnNNalSSuSxt | 0 | 0 | 1 |
| AmAprCCipCnNNalSSuSxtT | 0 | 0 | 1 |
| AmAprCCnNalSSuSxtT | 0 | 0 | 2 |
| AmAprCCnNalSuSxtT | 0 | 0 | 1 |
| AmAprCCnNNalSSuSxt | 0 | 0 | 1 |
| AmAprCCnNNalSSuSxtT | 0 | 0 | 8 |
| AmAprCCnNNalSuSxtT | 0 | 0 | 1 |
| AmAprCCnNSSuSxtT | 0 | 0 | 44 |
| AmAprCCnSSuSxtT | 0 | 0 | 24 |
| AmAprCCnSSuT | 1 | 0 | 1 |
| AmAprCnFrSSuSxt | 0 | 0 | 1 |
| AmAprCnNNalSuSxtT | 0 | 0 | 1 |
| AmAprCnNSSuSxt | 0 | 0 | 1 |
| AmAprCnNSSuSxtT | 0 | 0 | 2 |
| AmAprCnSSuSxtT | 4 | 5 | 38 |
| AmAprCnSSuT | 0 | 1 | 26 |
| AmAprCnSuSxtT | 0 | 0 | 1 |
| AmAprCSSuSxtT | 0 | 0 | 2 |
| AmAprCSxtT | 0 | 0 | 1 |
| AmAprSSuSxtT | 0 | 0 | 1 |
| AmCCazSSuT | 0 | 0 | 1 |
| AmCCfNalSSuSxtT | 5 | 0 | 0 |
| AmCCfNalSSuT | 13 | 0 | 0 |
| AmCCfNSSuSxtT | 0 | 0 | 1 |
| AmCCfSSu | 1 | 0 | 0 |
| AmCCfSSuSxtT | 25 | 1 | 3 |
| AmCCfSSuT | 33 | 7 | 10 |
| AmCCipNNalSSuSxtT | 0 | 0 | 8 |
| AmCCnSSuSxtT | 0 | 0 | 1 |
| AmCfNalSu | 1 | 0 | 0 |
| AmCFrNalSuSxtT | 0 | 0 | 1 |
| AmCFrNalSuT | 0 | 1 | 0 |
| AmCFrSSuSxtT | 0 | 0 | 2 |
| AmCfSSuSxtT | 0 | 0 | 2 |
| AmCfSu | 1 | 0 | 0 |
| AmCipNalSuSxtT | 0 | 0 | 1 |
| AmCipNNalSSuSxtT | 1 | 0 | 0 |
| AmCNalSSuSxtT | 0 | 1 | 2 |
| AmCNalSSuT | 21 | 3 | 10 |
| AmCNalSu | 1 | 0 | 0 |
| AmCNalSuSxtT | 0 | 0 | 2 |
| AmCNNalSSuSxtT | 0 | 0 | 5 |
| AmCNNalSSuT | 0 | 0 | 3 |
| AmCNNalSuSxt | 0 | 0 | 1 |
| AmCNSSuSxt | 0 | 0 | 1 |
| AmCnSSuSxtT | 0 | 0 | 1 |
| AmCNSSuSxtT | 0 | 1 | 68 |
| AmCnSSuT | 0 | 0 | 1 |
| AmCNSSuT | 0 | 1 | 2 |
| AmCNSuSxt | 0 | 0 | 1 |
| AmCNSuSxtT | 1 | 0 | 1 |
| AmCNSuT | 0 | 0 | 1 |
| AmCNT | 0 | 0 | 1 |
| AmCSSu | 0 | 0 | 1 |
| AmCSSuSxt | 0 | 0 | 20 |
| AmCSSuSxtT | 62 | 9 | 798 |
| AmCSSuT | 319 | 26 | 75 |
| AmCSSxt | 0 | 0 | 1 |
| AmCST | 1 | 0 | 0 |
| AmCSuSxt | 0 | 0 | 72 |
| AmCSuSxtT | 23 | 1 | 5 |
| AmCSuT | 46 | 0 | 5 |
| AmCSxtT | 1 | 0 | 1 |
| AmCT | 1 | 0 | 1 |
| AmFrNSu | 1 | 0 | 0 |
| AmFrSuSxtT | 0 | 0 | 1 |
| AmNalSSuSxtT | 1 | 0 | 0 |
| AmNalSSuT | 0 | 0 | 2 |
| AmNalSu | 2 | 0 | 0 |
| AmNalSuSxtT | 1 | 0 | 35 |
| AmNNalSuSxtT | 1 | 0 | 9 |
| AmNSSu | 1 | 0 | 0 |
| AmNSSuSxtT | 0 | 0 | 10 |
| AmNSSuT | 0 | 0 | 2 |
| AmNSuSxtT | 2 | 0 | 2 |
| AmSSu | 20 | 1 | 43 |
| AmSSuSxt | 2 | 0 | 0 |
| AmSSuSxtT | 5 | 1 | 110 |
| AmSSuT | 188 | 40 | 364 |
| AmST | 1 | 0 | 0 |
| AmSu | 6 | 5 | 5 |
| AmSuSxtT | 16 | 5 | 29 |
| AmT | 3 | 1 | 2 |
| AprCCnNalSSuSxtT | 0 | 0 | 2 |
| AprCCnSSuSxtT | 0 | 0 | 4 |
| AprCnSSuT | 0 | 0 | 2 |
| AprCnT | 0 | 0 | 1 |
| AprT | 0 | 0 | 1 |
| C | 0 | 0 | 1 |
| CCfFrSSuT | 0 | 0 | 1 |
| CCipNNalSSuSxtT | 0 | 0 | 1 |
| CCnSSuSxtT | 0 | 0 | 1 |
| CipNalSSuT | 7 | 0 | 0 |
| CipSSuT | 5 | 0 | 0 |
| CNalSSuSxtT | 0 | 0 | 3 |
| CNSSuSxtT | 0 | 0 | 1 |
| CSSu | 0 | 0 | 1 |
| CSSuSxt | 0 | 0 | 1 |
| CSSuSxtT | 0 | 0 | 39 |
| CSSuT | 3 | 0 | 0 |
| CSu | 0 | 0 | 1 |
| CSuSxt | 0 | 0 | 1 |
| CSuSxtT | 0 | 0 | 2 |
| CSuT | 3 | 0 | 0 |
| CT | 0 | 0 | 1 |
| FrSuSxt | 0 | 0 | 1 |
| FrSuSxtT | 0 | 0 | 1 |
| NalSSu | 0 | 0 | 1 |
| NalSSuT | 3 | 0 | 3 |
| NNalT | 0 | 0 | 1 |
| NT | 0 | 0 | 2 |
| S | 6 | 3 | 4 |
| sensitive to all tested antimicrobials | 163 | 118 | 42 |
| SSu | 17 | 2 | 9 |
| SSuSxt | 0 | 1 | 0 |
| SSuSxtT | 0 | 0 | 3 |
| SSuT | 10 | 4 | 18 |
| Su | 2 | 0 | 1 |
| SuSxt | 1 | 0 | 3 |
| SuSxtT | 0 | 0 | 20 |
| SuT | 1 | 0 | 1 |
| T | 82 | 8 | 100 |
